# Causes and differentials of childhood mortality in Iraq

**DOI:** 10.1186/1471-2431-9-40

**Published:** 2009-06-22

**Authors:** Naira A Awqati, Mohamed M Ali, Nada J Al-Ward, Faiza A Majeed, Khawla Salman, Mahdi Al-Alak, Naeema Al-Gasseer

**Affiliations:** 1 Independent Consultant, Amman, Jordan; 2 World Health Organization, Eastern Mediterranean Regional Office (EMRO), Cairo, Egypt; 3 World Health Organization/Jordan Country Office, Amman, Jordan; 4 World Health Organization/Iraq office, Amman, Jordan; 5 Department of Maternal and Child health, Ministry of health United Arab Emirates, Abu Dhabi, United Arab Emirates; 6 Central Organization for Statistics & Information Technology, Ministry of planning, and development cooperation, Baghdad, Iraq

## Abstract

**Background:**

Limited information is available in Iraq regarding the causes of under-five mortality. The vital registration system is deficient in its coverage, particularly from rural areas where access to health services is limited and most deaths occur at home, i.e. outside the health system, and hence the cause of death goes unreported. Knowledge of patterns and trends in causes of under-five mortality is essential for decision-makers in assessing programmatic needs, prioritizing interventions, and monitoring progress. The aim of this study was to identify causes of under-five children deaths using a simplified verbal autopsy questionnaire.

The objective was to define the leading symptoms and cause of death among Iraqi children from all regions of Iraq during 1994–1999.

**Methods:**

To determine the cause structure of child deaths, a simplified verbal autopsy questionnaire was used in interviews conducted in the Iraqi Child & Maternal Mortality Survey (ICMMS) 1999 national sample. All the mothers/caregivers of the deceased children were asked open-ended questions about the symptoms within the two weeks preceding death; they could mention more than one symptom.

**Results:**

The leading cause of death among under-five children was found to be childhood illnesses in 81.2%, followed by sudden death in 8.9% and accidents in 3.3%. Among under-five children dying of illnesses, cough and difficulty in breathing were the main symptoms preceding death in 34.0%, followed by diarrhea in 24.4%. Among neonates the leading cause was cough/and or difficulty in breathing in 42.3%, followed by sudden death in 11.9%, congenital abnormalities in 10.3% and prematurity in 10.2%. Diarrhea was the leading cause of death among infants in 49.8%, followed by cough and/or difficulty in breathing in 26.6%. Among children 12–59 months diarrhea was the leading cause of death in 43.4%, followed by accidents, injuries, and poisoning in 19.3%, then cough/difficulty in breathing in 14.8%.

**Conclusion:**

In Iraq Under-five child mortality is one of the highest in the Middle East region; deaths during the neonatal period accounted for more than half of under-five children deaths highlighting an urgent need to introduce health interventions to improve essential neonatal care. Priority needs to be given to the prevention, early and effective treatment of neonatal conditions, diarrheal diseases, acute respiratory infections, and accidents. This study points to the need for further standardized assessments of under-5 mortality in Iraq.

## Background

Children are the promise and the future of every nation, being the core of its development. Investing in children's health and development means investing in the future of a nation. Children are also a vulnerable group whose needs and rights must be protected, including the right to health and development [1]. Child mortality is a critical measure of the wellbeing of children and a good proxy indicator of the overall level of development [2]. Several generations of Iraqi children born since the early 1980s have faced adverse conditions which affected their nutrition, health, and mortality rates negatively. Knowledge of the causes of child mortality in Iraq is necessary to draw up strategies and plans to reduce child mortality in order to reach the global millennium development goals.

Iraq has a young population; 45% are under the age of 15 years, and those below 5 years of age constitute 17% of the population (3.9 million); two-thirds of the population lives in urban areas [3]. In 1999, UNICEF, in partnership with the Government of Iraq and the local authorities in Kurdistan Region, carried out two parallel household surveys to estimate the levels, trends and differentials of childhood mortality and the overall maternal mortality ratio [2]. In this paper the findings of our in-depth analysis of the results of the survey for childhood mortality of under-five year old by selected socio-demographic characteristics, and the causes of death are presented.

## Methods

Two cross-sectional household surveys were conducted in the South/Center and Kurdistan regions in February-March and April-May 1999, respectively. Altogether 40,477 married women between 15 to 45 years were interviewed in 23,978 households in the South/Center and 16,499 in Kurdistan by trained interviewers using a structured questionnaire that was developed using the Demographic and Health Surveys (DHS) questionnaire [2]. The questionnaire drew upon worldwide experience of deriving childhood mortality estimates by using relevant parts of the DHS-core questionnaire, with modifications that adapted it to the local circumstances.

The sample was designed to provide representative estimates of child mortality by region, key socio-economic and bio-demographic characteristics (such as place of residence, mother's education level, sex of the child, age of the mother at birth, birth order, preceding birth interval and consanguinity of parents). The data from the two surveys were merged and weighted to give national estimates for childhood mortality rates in Iraq. The following indices of childhood mortality were estimated: neonatal, post neonatal, infant, child and under-five mortalities.

Detailed information on the survey design, the questionnaire, and the training of the filed staff can be found in an earlier paper [2]. The birth history section of the questionnaire was the source for the information used to derive mortality estimates; it began with questions on the number of sons and daughters living with the mother in the order of their birth, those who lived elsewhere and those who have died. These questions were followed by retrospective birth history, in which the respondent was asked to list each of her births, beginning with the first birth, survivorship status, and current age or age at death for each of the respondent's live births. Information on living children was verified by requesting the civil identification card of each child, from which the exact date of birth was recorded.

In the case of dead children during the period 1994–1999 the mother/care taker was asked (using open-ended questions) about symptoms within the last two weeks before death, and leading up to death, the questions were based on a verbal autopsy questionnaire, and on the mother's/care taker perception of the cause of death. At the start, the mother was asked if the child had died because of illness, accident, or sudden death. In the case of death because of illness, she was asked about the symptoms in the last two weeks before death; the symptoms referred to diarrhea, vomiting, cough/difficult breathing, fever, rash, convulsions, congenital anomalies and other causes. In the case of sudden death, it was stressed during the training of the interviewers that the child should have had no symptoms of illness whatsoever prior to death. The protocol used had the capability of diagnosing multiple causes for the same death. Accidents included deaths because of injuries, traffic accidents, violence, poisoning, falling from heights, burning and drowning.

**Figure 1:**
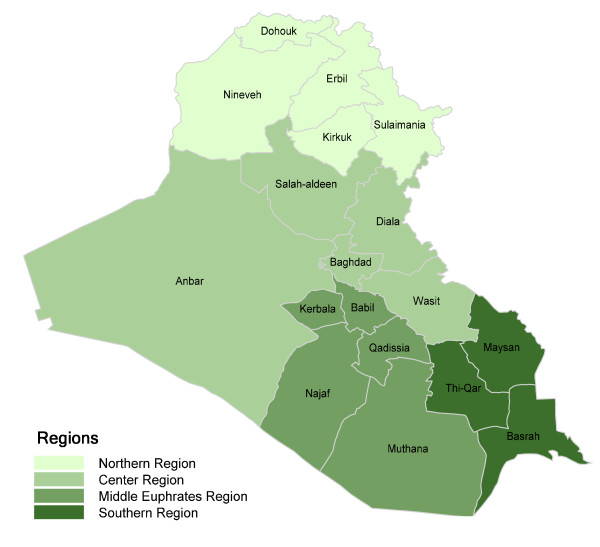
**Map of Iraq Regions/administrative grouping 1999**.

For the purpose of this paper, the geographical division of the country adopted at the time of the survey was as follows; Iraq was divided into four regions. The northern region included (Kirkuk, Erbil, Sulaimania, Dohouk, and Nineveh) governorates, the center region included (Baghdad, Diala, Wasit, Salah-aldeen, and Anbar) governorates, the middle Euphrates region included (Babil, Kerbela, Najaf, Qadissia, and Muthana) governorates, and the southern region included (Basrah, Thi-Qar and Maysan) governorates (figure 1). The analysis was based on the pooled weighted data and the methods of estimating childhood mortality rates were previously described by Ali et al [2].

## Results

A total of 4912 deaths of under-Five children were reported in the ICMMS 1999 during the five year period before the survey, 87.6% of the deaths, their cause of deaths or the symptoms preceding the deaths were recalled by their mothers/caretakers.

Table 1 show that during the years 1994–1999, death rates in rural areas exceed those of urban areas with 138.7 and 115.7 per 1000 live birth respectively. The southern region has the highest mortality rate in the country 144.1/1000 live birth while the north region has the lowest mortality rate for under-five children 100.8/1000 live birth.

**Table 1:** Number of birth and Under-5 mortality rates, by socio-demographic characteristics, during 1994-1999, all Iraq

	Demographic variables	Mothers		Live births	Under-5 mortality rate, per 1,000 live birth
		
		n	(%)	n	
Total		22,489	(100)	46,956	124.5
Place of residence					
	Urban	19,325	(85.9)	28,577	115.7
	Rural	3,164	(14.1)	18,379	138.7
					
Region					
	North	6,039	(26.9)	12,159	100.8
	Middle	8,981	(39.9)	18,637	125.8
	Middle Euphrates	3,894	(17.3)	8,224	136.8
	South	3,575	(15.9)	7,936	144.1
					
Level of education					
	No education	7,020	(31.2)	15,156	129.3
	Primary	10,718	(47.7)	22,690	129.6
	Intermediate	1,865	(8.3)	3,634	107.7
	secondary	1,506	(6.7)	2,915	111.8
	University	1,381	(6.1)	2,559	89.6
					
Sex of the child					
	Boy	11,657	(51.8)	24,225	128.8
	Girl	10,832	(48.2)	22,731	120.0
					
Preceding birth interval					
	First child	5,057	(22.5)	9,285	125.3
	<18 month	3,407	(15.2)	10,105	161.2
	18-35 months	8,137	(36.2)	18,804	101.1
	35+ months	5,888	(26.2)	8,762	111.5

Children born to mothers who never attended school had a mortality rate of 129.3 per 1,000 live births during the five years preceding the survey, which was similar to the rate for children of mothers with primary education 129.6 per 1,000 live births; however, the rate for children of mothers with secondary education was 111.8 per 1,000 live births.

More under-five deaths occurred among male children. Differences between male and female mortality rates were evident; higher mortality rates among males (128.8 per 1,000 live births) were found compared with female children (120.0 per 1,000 live births) during the years 1994–1999.

A birth interval of less than 18 months carries with it the highest risk of death for under-five children (161.2 per 1,000 live births), the least risk of death for under-five children occurred with preceding birth interval of 18–35 months (101.1 per 1,000 live births), followed by a birth interval of more than 35 months (111.5 per 1,000 live births), Table 1.

### Underlying causes of childhood deaths

Attempts were made to conduct verbal autopsy interviews with all mothers/caretakers of dead children. The results showed major differences in the cause of death structure by age group. During the years 1994–1999, illness was the leading cause of death in children under-five years of age (81.2%), with the highest percentage (90.3%) among infants 1–11 months of age, and 77.0% of neonatal deaths and 76.5% of deaths among children between 1–4 years. Deaths caused by illness were subdivided according to the symptoms two weeks preceding death, as reported by the child's mother/caretaker. In the neonatal period difficult breathing was the leading symptom preceding the death event (42.3%), in the infancy period, diarrhea was the leading cause of death as reported by the mothers (49.8%), followed by the symptoms of cough and/or difficult breathing (26.6%), then vomiting and fever (5.0% and 5.1%, respectively). In the 12–59 months age group, diarrhea was the leading cause of death (43.4%), followed by cough/difficult breathing (14.8%), then fever (5.9%). Table 2

**Table 2:** Percentage distribution of causes of death by age at death, region of residence and sex of the child, 1994-99

	**Age at death (months)**			**Region**				**Sex**		**Total**
		
	first month	1–11	12–59	North	Middle	Middle Euphrates	South	Male	Female	
Number of deaths (n)	(2744)	(1600)	(568)	(1029)	(1974)	(937)	(972)	(2645)	(2267)	(4912)
**Accident, injuries and Poisoning**	0.8	1.9	19.3	1.3	4.1	3.4	3.7	3.4	3.2	3.3
**Sudden death**	11.9	5.8	2.5	17.8	5.1	7.2	8.6	9.3	8.4	8.9
**Congenital malformation**	10.3	2.0	1.7	3.9	6.1	9.2	7.8	5.6	7.7	6.6
**Illness**										
Diarrhea	5.7	49.8	43.4	26.3	23.3	24.5	24.8	23.6	25.4	24.4
Vomiting	4.5	5.0	5.6	5.7	5.7	3.7	3.0	5.1	4.4	4.8
Cough/difficult breathing	42.3	26.6	14.8	29.1	36.7	35.1	32.7	33.2	35.0	34.0
Fever	2.8	5.1	5.9	3.9	4.2	3.0	4.2	4.3	3.4	3.9
Rash	0.2	0.9	0.5	0.1	0.9	0.3	0.1	0.4	0.5	0.5
Convulsion	1.3	0.5	1.3	2.2	0.8	0.4	0.9	1.1	1.0	1.1
Other illness										
Endocrine nutrition/metabolism,	0.9	0.8	1.3	2.4	0.6	0.6	0.1	1.0	0.8	0.9
Infectious & parasitic diseases	0.2	0.0	0.5	0.1	0.3	0.0	0.1	0.1	0.3	0.2
Oncology, Hematology/	1.4	0.4	0.7	0.9	0.8	0.9	1.8	1.3	0.8	1.0
Central nervous, Digestive, Jaundice, Uro-genital diseases	1.9	0.2	0.2	0.3	0.7	2.2	1.8	1.4	0.8	1.1
Disorders of pregnancy, difficult labor & perinatal conditions	3.6	0.2	0.0	2.0	1.7	3.3	1.8	2.9	1.1	2.1
Prematurity,	10.2	0.2	0.2	2.8	7.6	5.2	5.8	6.0	5.5	5.8
Unknown	2.0	0.8	2.1	1.3	1.5	1.0	2.7	1.4	1.8	1.6

Deaths due to illness were more prevalent in urban than in rural areas (57.5% and 42.5%, respectively). Deaths due to accidents were more common in rural areas than in urban areas (5% versus 3.2%), while sudden death was higher in urban than in rural areas (8.8% versus 7.7%). Figures by urban rural are not shown in the table.

Children living in the middle region died because of illness at a higher rate (84.7%) than children living in the northern region (77.0%). Little difference was observed between male and female mortality because of illness.

Sudden death was the second cause of deaths (8.9%), being most prevalent during the first few months of life. The highest percentage was found in the neonatal period (11.9%), with only 5.8% during the infancy period. Males were affected by sudden death more than females 9.3% versus 8.4%.

Accidents, injuries and poisoning constituted 3.3% of the total causes of deaths in all age groups, with the highest percentages found in the 12–59 months age group (19.3%; Table 2). More males died because of accidents than females (3.4% versus 3.2%), the highest death rate because of accident being found in the Middle region.

Congenital malformations led to 6.6% of deaths among under-five children with prevalence during the first month of life (10.3% of deaths). Children in the middle Euphrates died because of congenital malformation more than in other regions of the country.

## Discussion

As reported elsewhere, in Iraq, under-five mortality rate decreased from 87.8 per 1,000 live births in the second half of the seventies to 58.9 per 1,000 live births in the second half of the eighties, then increased again in the first and second halves of the nineties to (91.0 and 121.7 per 1,000 live births, respectively), During the same period, differences between rural and urban childhood mortality rates were evident, mother's education was positively associated with under-5 mortality and male children had higher mortality rates than female, specially among the neonates [4-6]. In the neonatal period, males had higher mortality rates than females (ICMMS), which is consistent with studies in various parts of the world [6]. Mortality rates for males and females are comparable in the post-neonatal and child age group.

Little is known from conventional sources about the epidemiology and causes deaths in children younger than 5 years of age in Iraq. The Civil Registration System, though extensive, records very incomplete information about births and deaths, especially in rural areas. In a study of the vital registration system in Iraq in 2002, only 15% of under-five children's deaths were registered [7], and, to our knowledge, no studies have been taken in the country from nationally representative samples on causes of child death. Determining the leading causes of death as accurately as possible and also determining the age groups at greatest risk of death are fundamental steps in making a community diagnosis that is, of determining the most frequent, serious, readily treatable or preventable conditions within the community, and there after planning an effective program to improve child survival [8].

Verbal autopsy is a technique "of growing importance" for estimating the cause-of-death distribution in populations without vital registration or other medical death certification. It involves collecting information about symptoms (including signs and other indicators) from the caretakers of each of a randomly selected set of deceased in some population of interest, and inferring the cause of death. Verbal autopsy studies are now widely used throughout the developing world to estimate cause-specific mortality [9]. The verbal autopsy represents a feasible approach to making it easier to compare findings and reduce subjectivity involved in assessing causes of death [10-12]. The instrument could be made more accurate if adapted based on a mothers' recognition of the signs and symptoms, as well as the terms they use to describe them [13]. Kalter et al. [14] developed and validated the verbal autopsy methods to diagnose several causes of neonatal illness and deaths in developing countries. A recent WHO publication presented a standard verbal autopsy method which was validated in three countries [15]. With verbal autopsy studies, the sensitivity and specificity of diagnosing diarrhea are in the range of 36% to 96%, and for acute lower respiratory infections are 28% to 91% [16]. In spite of the limitations of the verbal autopsy approach for ascertaining cause of death in impoverished settings, it remains the best available.

We found major differences in the cause of death structure by age group. In children younger than 5 years old, the largest single category of deaths was associated with acute respiratory illness (34.0%), the second largest category was diarrhoea (24.4%), the two causes together accounting for nearly half of all children's' deaths in Iraq. Sudden death (8.9%) was the next major cause of death followed by prematurity (5.8%) and accidents (3.3%).

The proportional distribution of deaths in children less than 5 years in the WHO Eastern Mediterranean Region (EMRO) estimates of causes of death among children indicate that neonatal deaths constitute 43% among under-five children deaths, followed by pneumonia (21%) and diarrhea (17%) [17]. In our analysis for the five years period preceding the survey, neonatal mortality accounted for 55.8% of the mortality in under-5 children, which is more than 47% reported in the EMRO region [18], but comparable to 50.1% reported in Egypt [16]**, as a**lso of 47% recorded in South East Asia [19], but it is higher than the global estimate of neonatal deaths (38%) [20]. The causes of death in children less than five years were studied in the Syrian Arab Republic using a structured verbal autopsy questionnaire. About 44% of deaths were among neonates [12]. We did not find measles a cause for death of under-five children, which is different from the regional estimates of measles deaths of 4%, and from the study from Bangladesh in which few deaths were identified as due to measles alone (0.7%) [11]. However, we do believe that most mothers/care takers in Iraq are able to diagnose the full-blown picture of measles; it is possible that some mothers may have missed cases of measles; however this finding could reflect a success story of the National Extended Program of Immunization in Iraq.

The high percentage of neonatal deaths could be explained by the deterioration of the health services and the socioeconomic conditions of the population at large during the nineties, especially that of women and children. All these factors had a deleterious effect on delivery and neonatal services. In 2001, an assessment of newborn care undertaken in 35 Maternal and child hospitals in Iraq showed that policies and guidelines on newborn care are non-existent. Only 46.7% of the doctors in these hospitals were trained on newborn resuscitation, with 51.6% receiving practical training for less than one hour on the procedure. Of the nurses working in the delivery rooms, 22.5% were trained on newborn resuscitation, and 36.7% of the medical and nursing staff still practiced holding the baby upside-down to stimulate breathing. Drying the baby with warm towels was practiced by only 13.5% of the staff working in delivery rooms [21].

Among neonates cough and/or difficult breathing accounted for 42.3% of neonatal deaths, these causes include pneumonia and respiratory distress syndrome. Other causes include congenital malformation (10.3%), prematurity (10.2%), sudden death (11.9%) and disorders of pregnancy and perinatal conditions (3.6%). These figures are different from the global estimates which indicate that causes of neonatal deaths are preterm birth (28%), severe infections including sepsis/pneumonia (26%), tetanus (6.5%), diarrhea (2.8%), complications of asphyxia (23%), and congenital abnormalities (7%) [19].

Preterm births as a cause for neonatal deaths was lower in our study than the global estimates [19], which could reflect the lower level of perception of women about prematurity; so is the figure for severe infections, which are both clinical diagnoses. However the high percentage of sudden deaths syndrome among neonates could not be explained, although particular attention to the definition of sudden death had been stressed during the training of the interviewers in the survey. In the Syrian study, sudden death was lumped with other defined causes (9%). In a study from Guinea on the causes of death among neonates, 9% were without diagnosis and 3% with no possible diagnosis [22]. Could some of these percentages reflect the sudden death syndrome? Classifying a large proportion of deaths as "early neonatal or pregnancy or delivery related" is not very helpful, making it very important that future studies attempt to classify these deaths further.

Globally, injury mortality is about 4 to 5% annually in most countries, and constitutes 5–10% of deaths among children less than five years, although the relative importance in the one to four year period is much greater (in the order of 25 to 35%) [23]. Deaths because of accidents injuries and poisoning ranked as the fifth major cause of under-five children deaths in the present study, contributing to 3.3% of under 5 deaths and 19.3% of deaths in the 12–59 age group, both figures are less than the global figures mentioned above, but comparable to the Regional figure of 3% reported by EMRO [17]. In a study by Baqui et al [11], accidents caused 8.8% of deaths among children younger than 5 years of age. This figure is more than twice that found in the present study, but the rate of 22.9% reported for the age group of 12–59 is comparable to the rate we have determined.

The causes in this category included deaths due to traffic accidents, drowning, burning, falling from heights, and poisoning by kerosene, other petroleum products, agricultural pesticides and household cleaners. It is important to recognize the burden of injury among young children in Iraq; most deaths following injury occur in the very hot summer because of poisoning with kerosene and petroleum products when thirsty toddlers drink petroleum products and detergents kept in drinking water cans in households. Fire is another important cause of death, especially in the cold winter months where unsafe kerosene heaters are used for warmth. Falls from unprotected roofs accessible for children is another important cause of death among children.

Globally congenital malformations contribute to 8% of neonatal deaths [17], which is less than our estimated rate of 10.3%. Congenital malformations are associated with flawed embryological development, the cause of most congenital malformations being unknown, although it is clear that good nutrition and prenatal care significantly reduce the number of malformed fetuses. Some congenital malformations result from genetic defects, others from environmental factors.

Congenital malformation rate in the middle Euphrates region is more than double that of the North region 9.2% versus 3.9% which could be explained by the widespread economic hardship and environmental factors which effected negatively the populations' health of this region, among which was the use of mustard gas during the 1991 gulf war [24]. In a large epidemiological study of tens of thousands of Gulf War veterans, they were two to three times more likely to have birth defects in their children than the general population [25]. The possibility of mustard gas fall-out on the geographical location of the Middle Euphrates is higher than the north region due to the proximity of the region to Hafir al Batin, where mustard gas had been detected in 1991.

The higher undr-5 mortality rate observed in the South and Middle Euphrates region compared to the North region is mainly due to the South region being on the forefront of the 8 years Iran-Iraq war and the 1991 Gulf War, compounded by the economic hardships of the region.

In the last 20 years of the twentieth century, the whole country experienced one of the most catastrophic economic declines in modern history; households' income witnessed a continuing decline; the GDP per capita has been declining since around 1980 due to a combination of wars, sanctions, and economic mismanagement. During the Iran-Iraq war real GDP per capita fell by an astonishing 57% between 1980 and 1988, and even further before the implementation of the UN's oil-for-food programme in 1996 [26].

Broad economic sanctions were applied on Iraq during the nineties of the last century; many studies documented the adverse effects of sanctions on the health and survivor of children [27,28]. Per capita spending on health fell dramatically; an analysis in 2003 by the Iraq Ministry of Health suggested that the funds available for health were reduced by 90% during the 1990s [29]. Sanctions resulted in higher rates of malnutrition especially among under-five children; the period was characterized by lack of medical supplies, and the spread of diseases from lack of clean water.

The dramatic changes in the child survival rates in southern central Iraq have been attributed to a variety of factors, including the declining economic well-being of the population, its effect on mother and child nutrition levels, as well as the declining access to healthcare and food security [30]. UNICEF in association with the UN's World Food Programme (WFP) reported that: "One out of every 4 Iraqi infants is malnourished. Chronic malnutrition among under-five children has reached 27.5% [31]. Historically, the Centre and South had better conditions than the North, but in the 1990s the situation reversed [29], after the implementation of the food-for-oil program in 1996. Of the oil revenues, 15% was earmarked for humanitarian supplies for the Kurdish population in northern Iraq, while ~25 cents per person per day for food and medicine was available for Central and Southern Iraq [32]. The north received 22% more per capita in dollar-value goods than the centre and south from the oil-for-food programme. The system of deductions resulted in per capita spending in the Center-South of only 61% that in the North until December, 2000 (later 69%), a very substantial difference. Moreover, the sanctions allowed the North to derive cash from 10% of its oil sales allocation [33]. More NGOs worked in the three governorates in Kurdistan (Sulaimania, Dohouk and Erbil) which contributed to improved health and nutrition of women and children in the region. All these factors acted concurrently to affect negatively under-five's health to a greater extent in the South region, while children's health in the north region benefited from the improved socio-economic conditions.

The Millennium Development Goals (MDGs) represent the widest commitment in history to addressing global poverty and ill health [34]. The Millennium Development Goal for child survival (MDG-4) to reduce childhood mortality by two-thirds between 1990 and 2015 will not be met without substantial reductions in neonatal mortality [35].

To develop effective strategies for reducing neonatal and under-five child mortality in the 21st century, studies of the underlying factors that contribute to morbidity and mortality of children in Iraq should be conducted. These studies must understand not only the biologic factors but also the social, economic, psychological, and environmental factors that contribute to maternal and infant deaths. A thorough review of the quality of health care, especially that of neonates, and access to care for all women and infants is needed, to avoid preventable mortality and morbidity, and to develop public health programs that can eliminate disparities in health [36].

Research and focused programs, especially for neonates, to enable mothers to identify acute lower respiratory infections, particularly pneumonia, and encourage timely and appropriate seeking of care, strengthening of acute lower respiratory infections case management at the primary care facilities being important priorities. Another priority is the systematic introduction of corticosteroid in premature birth, which can assist in the reduction of cases of Respiratory Distress Syndrome and hence deaths among neonates.

Introducing preventive programs to decrease mortality because of accidents in the age group of 1–4 is important. Innovative interventions to reduce childhood mortality from road accidents, drowning, burning, falls from heights and poisoning, should be designed and tested. While promotion of oral rehydration for diarrhea and antibiotic treatment for dysentery should continue. Broader preventive interventions including provision of safe water and sanitation, and improvements in personal hygiene require more attention. Further intensification of immunization programs is essential to reduce child mortality.

To be on the track for achieving the MDG-4, Iraq is facing very serious challenges, with the political instability, the ongoing – albeit reduced – violence which has hindered access to primary care in the south and middle region, and the insecurity which makes it difficult for people to travel to the services they need. Other constraints external to the health system include factors, such as the protracted failure of the electricity and the sanitation systems.

The overall policy environment and the political instability, are both inconsequential to materializing the MDGs in Iraq. However Iraq would be capable of a "dramatic" improvement in child survival and achieving the MDG to reduce U 5 mortality in the country if peace and political will prevailed.

## Limitation of the study

Malnutrition was not included in the questionnaire design, as well as questions about breast feeding and immunization status of deceased children. All of these have great effect on mortality in children. Steps and criteria for assigning causes of childhood deaths were not incorporated in the study design. Training in Kurdistan was done separately and by different facilitators

## Conclusion

Cause-of-death information and the epidemiogical facts are needed to provide evidence for health planning, and to prioritize the delivery of the necessary interventions to reduce child mortality. The information regarding causes of death in under-five children provided by this study can assist in guiding national plans and program interventions for reducing their mortality in Iraq; at the same time it will assist in assessing changes over time.

Our findings illustrate the urgent need for more comprehensive improvement of prenatal and postnatal care in Iraq, as well as the need for robust programs for combating respiratory, diarrheal diseases and accidents as the main causes of death among children younger than five. Children living in the Southern and Middle Euphrates region are at a higher risk of dying before reaching the age five, there is the need to emphasize interventions to reduce mortality in both regions.

## Competing interests

The authors declare that they have no competing interests.

## Authors' contributions

NAA, KS and NJA were principal investigators, and were involved in writing the survey protocol, participated in its design and coordination, supervised the survey implementation and analysis of the data. NAA drafted the manuscript. MMA was the principal statisticians who supervised all steps of data management, data analysis and drafted the manuscript. FAM, MA, and NA assisted in the analysis of data, paper design and in revising the manuscript, as well as providing critical comments.

## Pre-publication history

The pre-publication history for this paper can be accessed here:


